# Embracing Their Prey at That Dark Hour: Common Cuttlefish (*Sepia officinalis*) Can Hunt in Nighttime Light Conditions

**DOI:** 10.3389/fphys.2020.00525

**Published:** 2020-06-10

**Authors:** Melanie Brauckhoff, Magnus Wahlberg, Jens Ådne Rekkedal Haga, Hans Erik Karlsen, Maria Wilson

**Affiliations:** ^1^Department of Biology, University of Southern Denmark, Odense, Denmark; ^2^The Fisheries and Maritime Museum, Esbjerg, Denmark; ^3^Department of Biosciences, Faculty of Mathematics and Natural Sciences, University of Oslo, Oslo, Norway; ^4^NIRAS A/S, Aarhus, Denmark

**Keywords:** cephalopod vision, *Sepia officinalis*, cuttlefish, predatory behavior, low light vision

## Abstract

Cuttlefish are highly efficient predators, which strongly rely on their anterior binocular visual field for hunting and prey capture. Their complex eyes possess adaptations for low light conditions. Recently, it was discovered that they display camouflaging behavior at night, perhaps to avoid detection by predators, or to increase their nighttime hunting success. This raises the question whether cuttlefish are capable of foraging during nighttime. In the present study, prey capture of the common cuttlefish (*Sepia officinalis*) was filmed with a high-speed video camera in different light conditions. Experiments were performed in daylight and with near-infrared light sources in two simulated nightlight conditions, as well as in darkness. The body of the common cuttlefish maintained a velocity of less than 0.1 m/s during prey capture, while the tentacles during the seizing phase reached velocities of up to 2.5 m/s and accelerations reached more than 450 m/s^2^ for single individuals. There was no significant difference between the day and nighttime trials, respectively. In complete darkness, the common cuttlefish was unable to catch any prey. Our results show that the common cuttlefish are capable of catching prey during day- and nighttime light conditions. The common cuttlefish employ similar sensory motor systems and prey capturing techniques during both day- and nighttime conditions.

## Introduction

Prey capture behavior of coleoid cephalopods have been described for several species (Wilson, 1946; Messenger, 1968; Hurley, 1976; Duval et al., 1984; Mendes et al., 2006). These studies suggest that decapodiform cephalopods employ similar visual hunting techniques, using either their eight arms, and/or their two fast-extendable tentacles to seize the prey.

In the common cuttlefish (*Sepia officinalis*) hunting can normally be divided into three distinct phases (Messenger, 1968): attention, positioning, and striking. The attention phase occurs when the common cuttlefish first becomes aware of a prey item. The eyes then fixate on the prey, and the body slowly turn such that arms and head are oriented toward the prey. During the positioning phase, it slowly moves closer to the prey until the predator-prey distance is approximately one mantle length (see Hanlon and Messenger, 2018). During this phase, the tentacles slowly extrude toward the prey. The movement of the tentacles remains under full motor control, and the orientation of the tentacles adjust to prey movements. The positive feedback from visual input and motor control is continuous during this “aiming” phase until the common cuttlefish enters the seizure phase. This phase is defined by the abrupt action when the tentacles shoot out with extreme speed in an *all-or-none* fashion (Messenger, 1968; Duval et al., 1984). Once the seizure phase has been initiated, the common cuttlefish loses any motor-control of the tentacles, and has no further ability to re-adjust their aim or speed (Messenger, 1968; Duval et al., 1984).

This rather “stereotypic” three-phase hunting strategy has been observed in all species of squid and cuttlefish where prey capture behavior has been studied (Messenger, 1968; Hurley, 1976; Duval et al., 1984; Mather and Scheel, 2014; Sykes et al., 2014). However, details of the hunting strategy may vary depending on prey type and agility (Duval et al., 1984). Fast-moving prey (e.g., shrimps or fish) are typically captured by a rapid final extrusion of the flexible tentacles. Slow-moving prey (like crabs) on the other hand, is largely caught by a final “jumping” method, where the cuttlefish seize the prey using their 8 arms (Duval et al., 1984; Villanueva et al., 2017). Zoratto et al. (2018) have in addition shown that intraspecific variations in hunting behaviors can be linked to “personality” differences between individual cuttlefish.

Decapodiform cephalopods in general have well developed eyes (Muntz, 1977; Hanlon and Messenger, 2018), and they are highly dependent on their vision during hunting (Young, 1963; Messenger, 1968; Hurley, 1976; Talbot and Marshall, 2011; York et al., 2016; Hanlon and Messenger, 2018). Interference with their visual system reduces hunting accuracy and success rate (Messenger, 1968; Chichery and Chichery, 1987). Since most cephalopods have just one visual pigment and thus one class of photoreceptors, they are considered to lack color vision (Hanlon and Messenger, 2018). However, they are highly sensitive to polarized light, and may use such cues during hunting (Messenger, 1981, 1991; Shashar et al., 2000; Pignatelli et al., 2011). The common cuttlefish have large and highly developed camera-type eyes which are capable of adapting to varying light levels. Common cuttlefish can rapidly adjust their pupil size in the range 100–3% of the maximal area (Douglas et al., 2005). Additional light/dark adaptation mechanisms documented in octopods, but so far, not specifically described in cuttlefish, include screening pigment migration, photoreceptor size modulation, and specialized foveas (see Hanke and Kelber, 2020).

The common cuttlefish lives from subtidal waters down to about 200 m, but are most abundant in the upper 100 m of the water column (Reid et al., 2005). Light conditions consequently vary a lot, ranging from high intensities near the surface on a sunny summer day (equivalent to terrestrial type conditions), to very dim light at deeper waters on a cloudy winter night. Natural illuminance at nighttime ranges from 0.0001 lux on a moonless overcast (starlight) night sky to around 0.002 lux on a moonless clear night sky with airglow (Schlyter, 2017), while a full moon on a clear night ranges from 0.05 to 0.3 lux (Kyba et al., 2017). These light intensities gradually diminish by depth in the ocean. In clear oceanic water, light drops by a factor of about 2.2% per meter (Curcio and Petty, 1951). Observations from the Atlantic Ocean have found light depletion of 3–7% per meter (Clarke and Wertheim, 1953), and in coastal waters with suspended particles such as: plankton, runoff from rivers and other impurities, and the light reduction can be even higher.

The common cuttlefish are known to be active during both day and night (Watanuki et al., 2000), and some of their physiological processes undergo circadian cycles in a way suggesting that physical activity may actually increase at night (Jaeckel et al., 2007). A study of the closely related giant Australian cuttlefish (*Sepia apama*) at their spawning grounds found that they ceased sexual signaling and reproductive behavior at dusk, and settled to the bottom to camouflage themselves against the background (Hanlon et al., 2007). Furthermore, similar observations of the common cuttlefish in the laboratory revealed that they do not only camouflage themselves at dusk, but also adapt their camouflage patterns to their surroundings at night (Allen et al., 2010). It has been proposed that common cuttlefish use this behavior to avoid predators with excellent night vision, and/or to increase their own hunting success at night (Allen et al., 2010). The fact that common cuttlefish can camouflage themselves successfully during night may reflect highly developed nighttime visual abilities (Warrant, 2007; Allen et al., 2010). However, to our knowledge, the kinematics of the rapid tentacular hunting technique of decapod cephalopods have only been described in daylight conditions. Therefore, it is currently unclear whether common cuttlefish readily hunt during nighttime conditions, and if so, whether they use the typical three-phase hunting strategy.

The tentacle seizure phase of squid and cuttlefish predatory behavior is too fast to be studied in any detail by the naked eye. Kier and Leeuwen (1997) therefore employed a high-speed film camera in order to describe the kinematics of this phase in *Loligo pealei*, and documented tentacle seizures reaching accelerations as high as 250 m/s^2^. Additional studies confirmed the tentacle striking to be quite stereotypic (Kier and Leeuwen, 1997). The high-speed frame-capture methodology introduced by Kier and Leeuwen is invaluable for fine-detail insights into the fast tentacle strikes in cephalopods.

Accordingly, in the present study, we used high-speed video recordings to evaluate the prey capturing techniques in individuals of common cuttlefish at different light levels. The examined light levels were day- and two nighttime conditions, as well as complete darkness. The purpose was to elucidate whether high versus low light conditions influenced the hunting technique and tentacle fast-seizure characteristics of common cuttlefish.

## Materials and Methods

### Experimental Animals

Five juvenile common cuttlefish with mantle lengths of 78 ± 7.6 mm (standard deviation) were used in the experiment. They were provided by Øresund Aquarium, University of Copenhagen, and kept in 70 l holding glass aquariums with a closed seawater system (salinity 33–35‰ and temperature 18–20°C). The common cuttlefish were fed mysids (*Praunus flexuosus*) and shrimps (*Crangon crangon, Palaemon adspersus*, and *P. elegans*) two times daily.

### Experimental Setup and Protocol

The experiment was carried out at Drøbak Marine Biological Station, University of Oslo, Norway. Common cuttlefish were tested in a transparent plexiglas aquarium (dimensions: 20 × 40 cm) with a water depth of 10 cm. Prey capture attempts were recorded under four light conditions, which we broadly refer to as daylight, moonlight, starlight night, and darkness below. We measured these light conditions in three ways. We used an Ocean Optics QE65000 spectrometer to describe the spectral distribution of the light sources, a Thorlabs PM100A Power meter to measure the gross flux of light over all wavelengths combined of the light sources and an Extech EA30 to make readings in lux. Lux is a standard measurement for light as perceived by the human eye. The spectral sensitivity of the human eye and eye of the common cuttlefish predominantly overlap, but common cuttlefish have a somewhat higher sensitivity for shorter wavelengths compared to human photopic vision, more similar to human scotopic spectral sensitivity. Conversions from irradiance to illuminance are only possible if the spectral distribution of the cuttlefish eye is compensated for. For the purposes in this study, spectral distributions of the light sources are biased toward longer wavelengths within the cuttlefish visual spectrum. We therefore argue that this bias will not produce any false positive results, since the vision of common cuttlefish peaks at shorter wavelengths and therefore will receive less light than actually reported in the different test conditions here. Experiments in daylight were conducted during the daytime, while testing in the other three light regimes were performed during the night to not disrupt the diel cycle of the test animals.

The test aquarium was shielded by a non-translucent box with a removable top. For daylight experiments, the top of the box was open, while it was closed during the three other light regimes. Three near-infrared lamps (model 995JH) provided sufficient light inside the box for high speed filming. Two of the IR-lamps were placed at one side of the aquarium and one at the opposite side.

The daylight experiments were conducted with natural light entering the experimental room through several large windows and with fluorescent ceiling lights (3000 K) turned on. Light intensities at the site of the experimental animal, i.e., inside the test aquarium and shielding box, and were approximately 200 lux. Even though natural sunlight was the main light source in the daylight experiments, the shielding box provided a distinctive reduction of the intensity, thus causing the relatively low daylight intensity of 200 lux. The lower light level, obtained by the shielding box, was deliberately chosen in order to reduce the stress of the animals and reduce the risk of affecting their eyes in ways that could potentially reduce their capacity to hunt in dimmer light conditions later.

**FIGURE 1:**
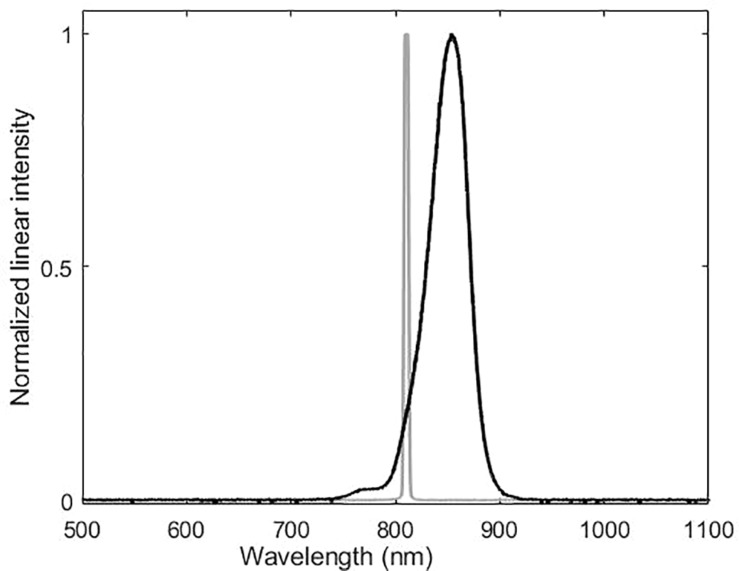
Spectral analysis of the near-infrared lamps used in the trials. Gray line is laser and black line LED. Peak intensity of the LED lamp is at around 850 nm. Approximately 1% of the intensity is between 700–765 nm.

Moonlight experiments were conducted with the laboratory ceiling lights off and with the experimental room shielded from ambient light. The three IR-lamps did not produce an even distribution of light in the test aquarium. The highest value, 0.3 lux, was obtained when the light sensor was directly facing the center of the IR-lamp at the typical “mysid –IR-lamp” distance of 35 cm during experiments. At other positions, the illuminance was around the Extech EA30 detection limit of 0.01 lux. We also measured the IR-lamps using the Thorlabs PM100A Power meter and the Ocean Optics QE65000 spectrometer. The light intensity peaked at 850 nm, in the infrared part of the spectrum, thus not detectable for humans and common cuttlefish. Low intensities, from approximately 0.5–1% of the entire energy spectrum, was found to be from 700–765 nm, which are partly within the cuttlefish visible spectrum (Figure 1). The three IR-lamps provided an irradiance of 3 W/m^2^, or less than 0.003 lux at the spectral range of the common cuttlefish. This corresponds to moonlight intensities in the cuttlefish visual spectrum from 380–740 nm.

Starlight experiments were performed similarly to the experiments in moonlight but adding a custom-made filter to the IR-lamps. The filters reduced the visual light to the extent that it was not possible to measure directly at relevant experimental distances (35 cm). Measurements very close to the IR-lamp revealed that the filter reduced the visual light by more than three orders of magnitude to an estimated 10^–6^ lux during the experiments at relevant distances.

For the experiments in darkness, the experimental set up was identical to the experiments in starlight with the only exception that the LED lamps were turned off and the only source of illumination was a near-IR laser (Olng 300 L). This laser was equipped with a custom-made filter to spread the laser beam. The laser has a very narrow spectral band (peak 810 nm) and very little stray light compared to the LED lamps. As common cuttlefish are not known to detect IR light, this experiment was performed in complete darkness from the common cuttlefish’ point of view.

The video camera was placed one meter above the aquarium providing a two-dimensional top view of the prey-capture sequence. Video recordings were performed using an IR-sensitive Mikrotron EoSens MC1362 camera and Inspecta-5 PCI-X frame grabber card. For day- and moonlight experiments we used 1,000 frames per second (fps), and for starlight and darkness 25 fps due to the limited available light. The lower frame rate during experiments in starlight and darkness did not allow for detailed, kinematic analyses. Monitoring of the test animal during experiments was done on a monitor connected to the high-speed camera.

Prey items (mysids, approximately 20 mm in length) were introduced into the aquarium through a small gate in the box. Prey were introduced with at least 15 min between each session. Test continued until the cuttlefish showed no more interest in the prey. Animals were used in only one trial sequence, and were kept in the experimental setup for a maximum of 24 h. The three different light settings were chosen in a random order in consecutive trials.

A total of 33 day-, 40 moon-, and 12 starlight prey capture events were recorded. In addition, 2 darkness trials were conducted. For the present study, 8 prey-capture sequences from daylight (with three different cuttlefish, *n* = 3), and 11 from moonlight experiments (with four different cuttlefish, *n* = 4) were selected for analysis using the following criteria: (1) Neither cuttlefish nor mysid were in touch with the side walls of the test tank; (2) Cuttlefish and mysid were at the same depth (near the bottom), assessed from visual inspection of the eye position of the cuttlefish; and (3) Cuttlefish tentacles were clearly visible during the entire prey capture sequence.

In addition, control trials were conducted to ensure that the cuttlefish did not rely on other sensory abilities than eyesight while hunting for prey under low light conditions. A live mysid was placed in a glass jar and sealed with a lid before it was placed in the test aquarium. In control trials, the cuttlefish was able to see the mysid, but all olfactory and mechanical/vibrational cues were eliminated. These controls were conducted during day- (*n* = 1) and moonlight (*n* = 1) conditions.

### Video Analysis

Video sequences were tracked in ImageJ (1.47) using the MTrackJ plugin (Meijering et al., 2012). Movements of the cuttlefish body, arms, and tentacles and the movement of the mysid were tracked on each video sequence in steps of 4 ms. During tentacle striking, tracking time steps were reduced to 1 ms in order to capture the very rapid tentacle movements during this phase. Tracking started 100 ms prior to the strike, and continued until 12 ms after the tentacles had made contact with the mysid.

The ImageJ tracking program provided raw numerical data for kinematic analysis. The distance between two tracking points was given by a basic distance formula. From the distances between tracking points, the velocity was calculated and smoothed over 5 consecutive measurements. Acceleration was calculated from the smoothed velocity data.

Statistical analysis was performed using GraphPad Prism version 8.0.2. Data from daylight and moonlight conditions were compared using a nested *t*-test, with the measurements from trials made with the same individual cuttlefish nested under each specimen.

## Results

### Prey Capturing Phases

**FIGURE 2:**
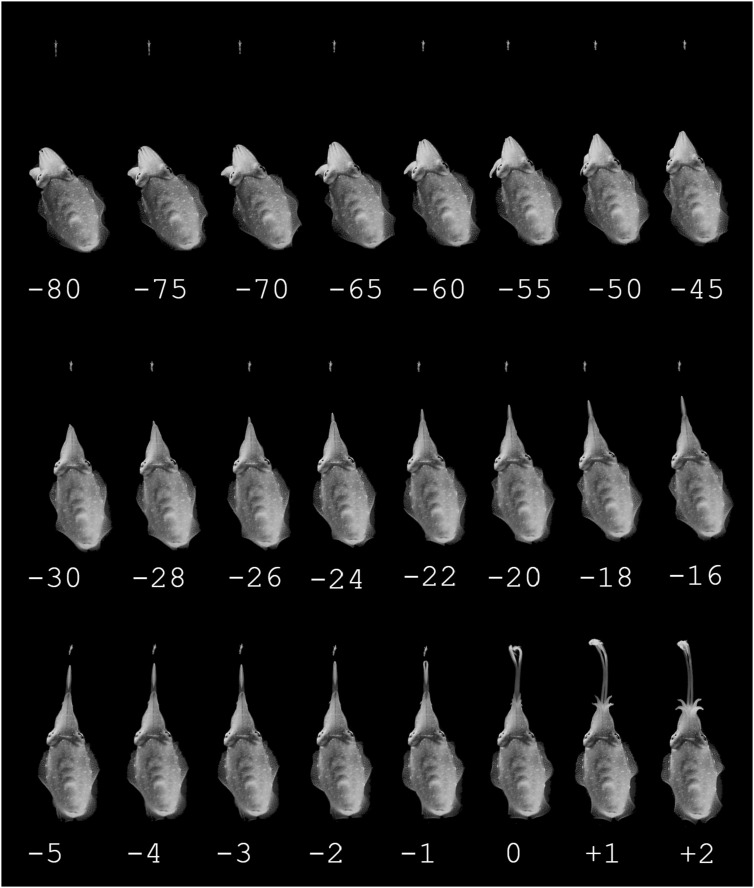
Example of a prey capture sequence of a cuttlefish hunting during moonlight conditions, the time in ms is stated below the pictures. Top row: Attention phase, starting by the cuttlefish detecting the prey and ending by having aligned the body axis in the direction of the prey. There are intervals of 5 ms between the pictures. Middle row: Positioning phase, starting with the cuttlefish slowly moving to the preferred distance to the prey, slowly ejecting the tentacles. There are intervals of 2 ms between the pictures. Bottom row: Seizure phase. The tentacles are ejected very rapidly in an all or nothing fashion. There is 1 ms between every picture.

**FIGURE 3:**
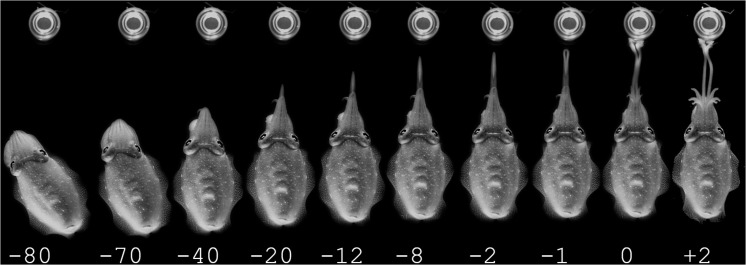
Prey capture experiments of cuttlefish with prey enclosed in a jar in moonlight conditions. The cuttlefish displays the same stereotypic behavior as when hunting free-swimming animals. The time in ms is stated below the pictures.

Every selected hunting sequence in day-, moon-, and starlight conditions roughly followed the same pattern in the way the cuttlefish changed its attention, positioning and finally seized the moving prey (Figures 2, 3).

The day- and moonlight trials showed no significant difference in all three phases of predatory behavior. During the attention phase (Figure 2A) the cuttlefish detected and fixated on the prey while the body moved to a position where the tip of the 10 arms faced the prey. During the positioning phase (Figure 2B), the cuttlefish slowly approached the prey while slowly extruding the tentacles with an average velocity of 0.13 m/s (SD ± 0.05, 19 video sequences from four cuttlefish; *n* = 4). The tentacles were kept closely together during the initial phase of the fast extension but opened in front to expose the suckers in the final phase when the tentacles made contact with the prey (Figure 2C). The outermost part of the tentacle tip stayed together (as visibly in Figure 2C frame 1). The split between the tentacles extended backwards toward the animal and was total when retraction of the prey began (Figure 2C).

During the experiments in starlight, the cuttlefish still readily performed typical three stage hunting and prey capture behaviors as illustrated in Figures 3A–C. Even though conditions of observations were significantly worse in these trials (due to the low light conditions and low frame rate), it was clear that fast tentacle seizure behavior involved an attention phase (Figure 3A) with the cuttlefish orienting toward the prey. Notably, all mysids were in their normal head up and tail down orientation, and thereby still in a visual behavior mode (see darkness section below). The attention phase in the cuttlefish was followed by a positioning phase (Figure 3B) and a tentacle seizure phase (Figure 3C). Overall, tentacle seizures in the starlight trials appeared very close to the day- and moonlight tentacle seizures, but reliable seizure velocity and acceleration values were not possible to obtain due the low frame rate.

In the darkness experiments, cuttlefish responded to the very low light levels with a complete inhibition of prey searching behavior and prey capture. Instead, they started to express a state of “panic-like” confusion by swimming uncontrolled around the aquarium. In addition, during these trials the mysid showed a clear change in their behavior by changing from the upright body position to a near horizontal body orientation and active swimming around the test tank. This was a behavior never seen in the day-, moon- and starlight trials. Thus, both the cuttlefish and the mysid shrimp showed distinctly changed behaviors when deprived of all visual light in the laboratory.

### Experimental Control Trials

**FIGURE 4:**
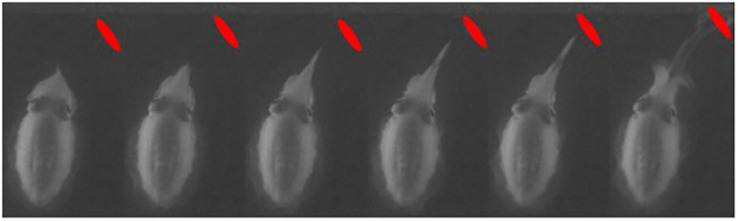
Cuttlefish catching an alive and free-swimming mysid during starlight conditions. The quality of the video is significantly reduced due to the very low light intensities. The location of the mysid is marked with a red dot. The cuttlefish displayed the same stereotypic hunting behavior as it did in day- and moonlight conditions.

Control trials contained a living mysid in a sealed glass jar. Both in day- and moonlight conditions, the cuttlefish were attacking the mysid in a similar way as when the mysid was swimming freely (Figure 4).

### Kinematics of Capturing Sequences

**FIGURE 5:**
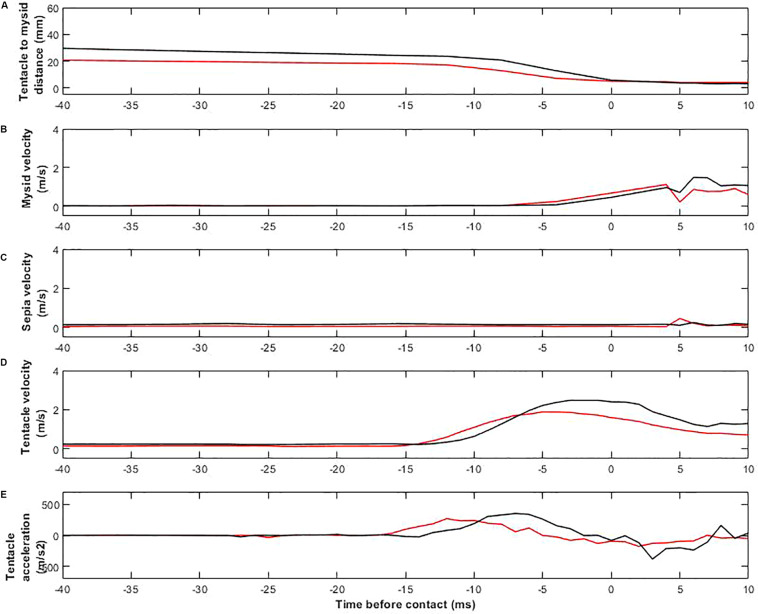
Plots of kinematics of prey capture strikes. Black lines represent daylight, red moonlight conditions. Time, *t* = 0, is defined as the video frame where the tentacle reaches the prey. **(A)** Distance between the tip of the cuttlefish’s tentacles and the prey’s head. **(B)** Velocity of the prey. **(C)** Velocity of the cuttlefish body. **(D)** Velocity of the tip of the tentacle. **(E)** Acceleration of the tip of the tentacle.

The detailed movements of cuttlefish and prey were very similar in day- and moonlight trials (Figure 5). Figure 5A shows the distance between the tip of the cuttlefish tentacle and the body of the prey during the positioning and seizure phases. The timing of the sequences is synchronized so that the instant when the tentacles touch the mysid is 0. The tentacular seizure occurred after an average of 88.0 ± 0.5 ms (8 video sequences from three cuttlefish, *n* = 3) in daylight, and 90 ± 1.6 ms (11 video sequences from four cuttlefish, *n* = 4) in moonlight trials. The distance between the tip of the tentacles and the prey (seizure distance) was 22.96 ± 3.3 mm (8 video sequences, *n* = 3) for seizures in daylight and 17.87 ± 4.0 mm (11 video sequences, *n* = 4) in moonlight. There was no significant difference between the mean time or distance upon tentacle seizure (nested *t*-test, *P* value = 0.08, df = 5, *t* = 1.8 and *p* = 0.13).

Before the tentacle made contact with the mysid, the velocity of the prey remained constant at 0.04 ± 0.01 m/s in both day- and moonlight trials. After contact, the mysid velocity increased rapidly to 0.9 ± 0.4 m/s (Figure 5B). Throughout the entire capturing sequence, the velocity of the cuttlefish body was approximately 0.13 ± 0.04 m/s (19 video sequences, *n* = 4) for both day-, and moonlight trials (Figure 5C).

When tentacle seizures were initiated, tentacle velocities and accelerations increased rapidly. The maximum velocity was 2.34 ± 0.27 m/s (8 video sequences, *n* = 3) in daylight and 2.03 ± 0.11 m/s (11 video sequences, *n* = 4) in moonlight (Figure 5C). The mean velocities were significantly different (Nested *t*-test, *P* value = 0.07, df = 5 and *t* = 2.3). The corresponding mean maximum accelerations were 418.3 ± 33 m/s^2^ (8 video sequences, *n* = 3) in daylight and 366.3 ± 18.5 m/s^2^ (11 video sequences, *n* = 4) in moonlight. The mean accelerations were significantly different (Nested *t*-test, *P* value = 0.03, df = 5 and *t* = 3.0).

## Discussion

The experiments showed that common cuttlefish prey capture behavior are not restricted by low light levels. The common cuttlefish successfully captured mysids during the three different simulated light levels; daylight, moonlight and starlight (Figures 2, 3, and 5), with very little difference in the common cuttlefish prey capture behavior between daylight and moonlight. The unchanged behavior during the starlight trial (Figure 4), combined with no successful prey captures and drastically changed behavior during the trials in complete darkness, indicate the importance of visual cues during predatory behavior.

Besides vision, cephalopods have very complex sensory systems, e.g., the sense of touch (Kier and Leeuwen, 1997; Hanlon and Shashar, 2003), and an olfactory organ (Polese et al., 2016). Earlier experiments indicate that common cuttlefish rely on mainly visual cues when hunting, since physically blinding cuttlefish resulted in a significant drop of attack rates (Messenger, 1968). It is unclear, however, to what extent Messenger’s (1968) results were confounded by behavioral changes in the blinded common cuttlefish, due to the rather brute-force method used. Still, our darkness experiments corroborates Messenger’s (1968) conclusions: When the common cuttlefish was deprived of any visual cues, they were not able to forage even though all other sensory cues were available.

During the daylight tests, common cuttlefish readily attacked the mysid shrimp employing the typical three-phase hunting strategy previously described in all other studied species of cuttlefish and many other species of decapodiform cephalopods (Figure 2; for an extensive review see Hanlon and Messenger, 2018). The maximum tentacle strike accelerations were similar, albeit sometimes higher, than the ones measured in *L. pealei* (see Kier and Leeuwen, 1997). The higher tentacle acceleration in common cuttlefish may indicate that this species is capable of catching faster moving prey than *L. pealei*, but this hypothesis needs to be validated in further experimentation. The tentacle velocity and acceleration were significant higher during day- than moonlight trials (Figure 5). Thus, even though the common cuttlefish performed similar hunting behaviors, there were some differences in the prey capturing techniques for our simulated day- and nighttime light levels.

Our results indicate that common cuttlefish might be able to navigate and forage at nighttime and in low-light environments. This strongly supports the notion that cuttlefish may perform advanced behaviors, including foraging and actively camouflaging themselves during nighttime (Watanuki et al., 2000). When all light cues were eliminated, the behavior of the common cuttlefish changed drastically, and did not seem to be able to catch any prey. We believe that a sensation of complete darkness in combination with a rather “sterile” aquarium, with no hiding places, might have been discomforting to the test animals, and that this was the cause of their altered behavior.

The behavior of the prey was similar in all trials, regardless of lighting. This implies that the movement of the prey did not affect the common cuttlefish approach during different light conditions. The body of the cuttlefish did not seem to alter its velocity throughout the entire attack sequence (Figure 5B). This might also indicate the stereotypic nature of the common cuttlefish’s hunting strategy. While the tentacles were slowly extruded toward the mysid, the rest of the cuttlefish body stayed motionless. The reason why this strategy is so efficient may be due to the prey keeping its attention on the cuttlefish body, missing the fact that the almost see-through tentacles are slowly approaching the prey.

The high catching performance of common cuttlefish in the low-light levels tested here strongly indicates that common cuttlefish can forage during very limited light conditions. The most important sensory stimulus used during foraging behavior are visual cues, even during very limited light conditions. Common cuttlefish may therefore be more active at night than what has previously been assumed. This may also explain why common cuttlefish actively adjust their camouflage during nighttime (Watanuki et al., 2000). We encourage studies in the wild, without the use of artificial light sources in the visible spectrum, to verify that even at that Dark Hour, tiny marine creatures might be both embracing death and being embraced – by our heroes: the true knights in the dwindling lights of the ocean nights (Shakespeare, 1626).

## Data Availability Statement

The datasets generated for this study are available on request to the corresponding author.

## Ethics Statement

All studies were conducted in accordance with the Norwegian Animal Welfare Act of 2018, the Regulation of Animal Experimentation of 2017 and approved as field studies at the Marine Biological Station Drøbak by the University of Oslo, Animal Welfare Unit (ref. 155 UiO – Department of Biosciences). All animals used in this study were treated according to the guidelines provided by the European Directive in Directive 86/609/EEC and Directive 2010/63/EU.

## Author Contributions

Experimental trials were conducted by MWi, JH, and HK. Data were analyzed by MB. Figures were made by MB and MWa. All authors contributed to the editoral work of this manuscript.

## Conflict of Interest

The authors declare that the research was conducted in the absence of any commercial or financial relationships that could be construed as a potential conflict of interest.
